# Metastatic spread of pleural mesothelioma to the peritoneum and gallbladder presenting as recurrent acute cholecystitis

**DOI:** 10.1093/omcr/omaf082

**Published:** 2025-06-27

**Authors:** Mina Fouad, Abed M Zaitoun, Dileep N Lobo

**Affiliations:** Nottingham Digestive Diseases Centre, Division of Translational Medical Sciences, School of Medicine, University of Nottingham, Queen’s Medical Centre, Derby Road, Nottingham NG7 2UH, Nottinghamshire, United Kingdom; NIHR Nottingham Biomedical Research Centre, Nottingham University Hospitals NHS Trust and University of Nottingham, Queen’s Medical Centre, Derby Road, Nottingham NG7 2UH, Nottinghamshire, United Kingdom; Nottingham Digestive Diseases Centre, Division of Translational Medical Sciences, School of Medicine, University of Nottingham, Queen’s Medical Centre, Derby Road, Nottingham NG7 2UH, Nottinghamshire, United Kingdom; NIHR Nottingham Biomedical Research Centre, Nottingham University Hospitals NHS Trust and University of Nottingham, Queen’s Medical Centre, Derby Road, Nottingham NG7 2UH, Nottinghamshire, United Kingdom; Nottingham Digestive Diseases Centre, Division of Translational Medical Sciences, School of Medicine, University of Nottingham, Queen’s Medical Centre, Derby Road, Nottingham NG7 2UH, Nottinghamshire, United Kingdom; NIHR Nottingham Biomedical Research Centre, Nottingham University Hospitals NHS Trust and University of Nottingham, Queen’s Medical Centre, Derby Road, Nottingham NG7 2UH, Nottinghamshire, United Kingdom; Perelman School of Medicine, University of Pennsylvania, 3400 Spruce Street, Philadelphia, PA, United States

**Keywords:** pleural mesothelioma, immunotherapy, cholecystitis, cholecystectomy, immunohistochemistry, check-point inhibitors

## Abstract

Malignant pleural mesothelioma, a rare, aggressive cancer primarily associated with asbestos exposure, is characterised by poor prognosis and limited treatment options. Distant metastases are uncommon, and peritoneal involvement is rare, while metastasis to the gallbladder is extremely unusual. We report the case of a patient with malignant pleural mesothelioma undergoing immunotherapy with nivolumab and ipilimumab, who developed recurrent acute cholecystitis. Imaging revealed features of acute cholecystitis with gallstones. She eventually underwent a laparoscopic cholecystectomy because immunotherapy could not be continued in the presence of infection. Histopathological and immunohistochemical analysis confirmed metastatic epithelioid mesothelioma. The gallbladder metastasis was likely haematogenous or from peritoneal seeding, rather than direct spread. This case underscores the importance of a high index of suspicion when evaluating atypical presentations of mesothelioma, particularly in the context of immunotherapy.

## Introduction

Malignant mesothelioma is a rare but aggressive malignancy arising from the mesothelium, with the pleura being the primary site in 90% of cases [1]. Asbestos exposure is the leading risk factor, implicated in up to 80% of cases, although other factors, including BRCA1-associated protein 1 (BAP1) mutations, ionizing radiation, and Simian Virus 40 (SV40), have also been associated with its development [2, 3]. Malignant mesothelioma is typically locally invasive, with a propensity to infiltrate adjacent structures. Distant metastases occur late in the course of the disease, with peritoneal involvement being rare, often presenting as ascites or painful complications such as bowel obstruction. However, autopsy studies suggest that distant metastases may be underdiagnosed [4]. Histologically, malignant mesothelioma is classified into three main subtypes: epithelioid (50%–70%), associated with a better prognosis; sarcomatoid (7%–20%), linked to the worst outcomes; and biphasic (20%–35%), which has features of both [5]. Rare variants, including the desmoplastic subtype, have also been described. Diagnosis relies on immunohistochemical staining, as histopathology alone is insufficient, and no single marker is definitive [5, 6]. We present the report of rare metastasis of a malignant pleural mesothelioma to the peritoneum and gallbladder, highlighting the diagnostic and therapeutic challenges associated with this atypical presentation.

## Case report

**Figure 1:**
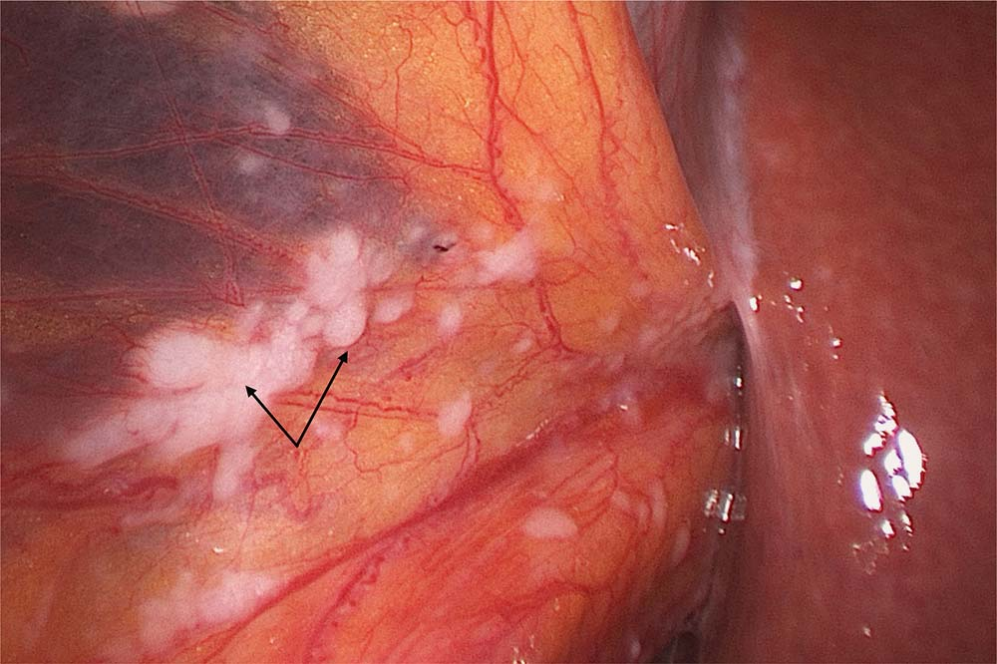
Laparoscopic view showing multiple nodules (arrows) on the falciform ligament.

**Figure 2:**
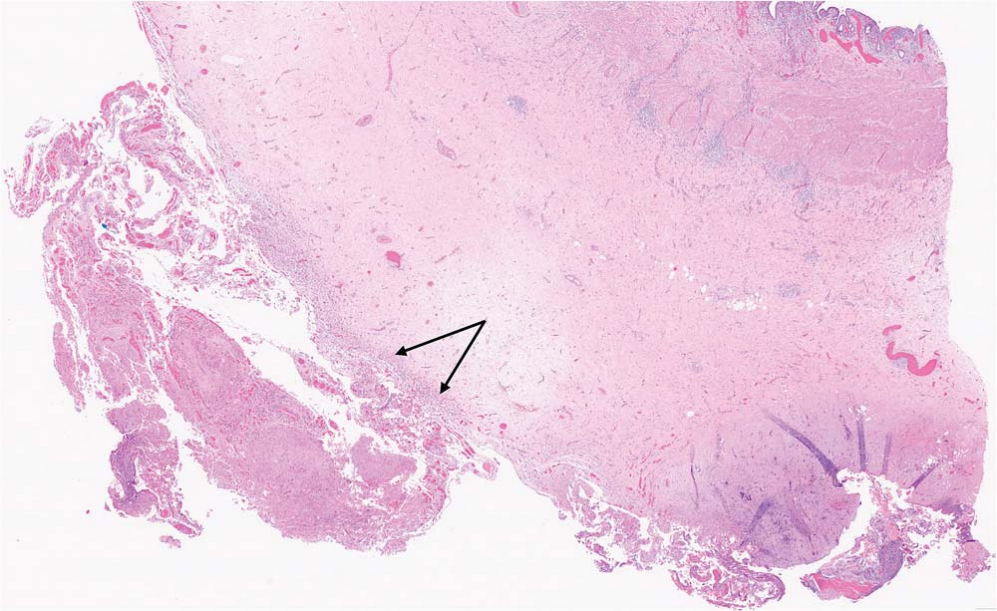
Section of gall bladder wall stained with haematoxylin and eosin showing malignant mesothelioma cells (arrows, 2× magnification) infiltrating the perimuscular tissue of the gall bladder.

**Figure 3:**
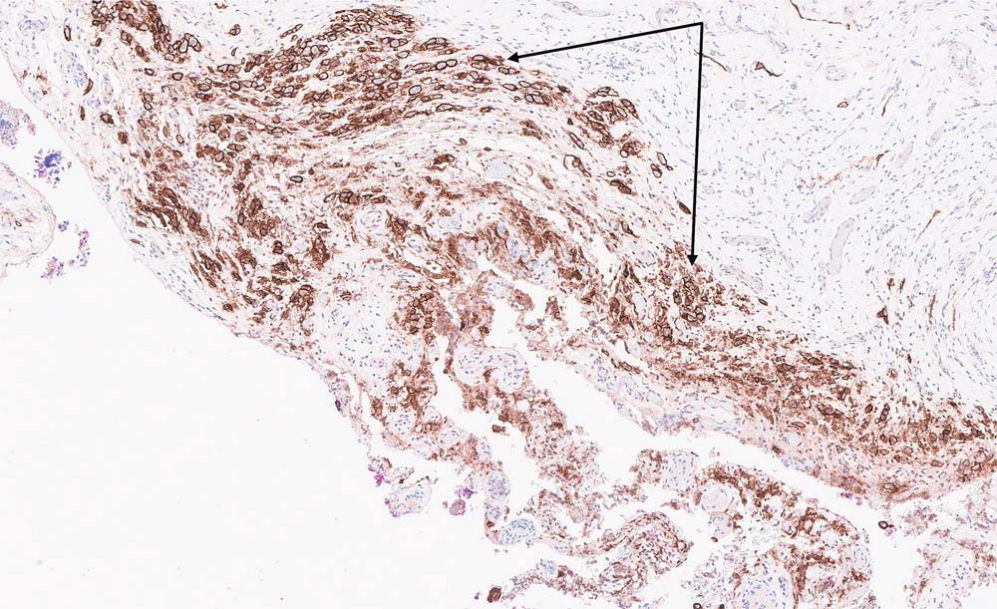
Tumour cells in the gall bladder stained positively with D2–40 (podoplanin), a marker for malignant mesothelioma, on immunohistochemistry (arrows, 10× magnification).

A 75-year-old woman on immunotherapy with the checkpoint inhibitors nivolumab and ipilimumab for pleural mesothelioma presented with recurrent acute cholecystitis. Computed tomography showed a large solitary gallstone in an inflamed, thick-walled gall bladder without perforation or a mass lesion. At each admission she was managed non-operatively with antibiotic therapy. As immunotherapy was discontinued because of infection, she eventually underwent laparoscopic cholecystectomy. Multiple miliary nodules were seen at laparoscopy on the peritoneum and falciform ligament (Fig. 1). The gall bladder was acutely inflamed without any visible nodules. Peritoneal biopsy showed neoplastic cells which stained positively on immunohistochemistry with D2–40 (podoplanin), WT1 (Wilms tumour-1), Cytokeratins 7 and 5/6 (CK7 and CK5/6) but negatively with BerEP4, consistent with metastatic malignant epithelioid mesothelioma. Haematoxylin and eosin stain of the gall bladder showed multiple foci of metastatic mesothelioma cells (Fig. 2) involving perimuscular fibrous tissue. Immunohistochemical staining with D2–40 (podoplanin) of the gall bladder showed similar staining as the peritoneal nodules (Fig. 3). The cystic duct margin and a lymph node were tumour free. The patient recovered and immunotherapy was recommenced. She was alive and well 8 months after the cholecystectomy.

## Discussion

Malignant pleural mesothelioma is a rare, aggressive cancer with a poor prognosis, with median survival ranging from 6 to 12 months and fewer than 5% of patients survive beyond five years [7]. It is usually a locally invasive tumour, and distant metastases are rare and often underdiagnosed, with peritoneal involvement typically presenting with ascites or bowel obstruction. Gallbladder metastasis is even more unusual and has not been previously reported [4, 8]. Current American Society of Clinical Oncology guidelines recommend staging CT scans of the chest and upper abdomen for all patients with malignant pleural mesothelioma, with further imaging of the lower abdomen and pelvis if distant metastases are suspected [9, 10]. This case presents a unique instance of malignant mesothelioma metastasising to both the peritoneum and gallbladder, undetected on preoperative imaging and without macroscopic gallbladder deposits intraoperatively, presenting significant diagnostic and therapeutic challenges.

The rarity of gallbladder involvement in malignant mesothelioma is due to the tumour’s primary pattern of local spread, with haematogenous or lymphatic dissemination occurring later [11]. In this case, the patient developed recurrent acute cholecystitis, initially managed conservatively, with imaging revealing gallbladder wall thickening but no mass lesion or nodules. The metastatic nature was confirmed postoperatively via histopathology, underlining the importance of thorough pathological assessment in atypical cases.

Immunohistochemistry was pivotal in diagnosis, with tumour cells in both peritoneal and gallbladder tissues staining positively for D2–40, WT1, CK7, and CK5/6, and negatively for BerEP4, confirming metastatic epithelioid mesothelioma. Given the absence of tumour involvement at the cystic duct margin or regional lymph nodes, gallbladder metastasis was likely hematogenous or due to peritoneal seeding rather than direct spread. This emphasises the need to consider mesothelioma in the differential diagnosis of unexplained gallbladder pathology in patients with malignant mesothelioma.

Immune checkpoint inhibitors like nivolumab and ipilimumab have revolutionized the treatment of malignant mesothelioma and shown efficacy in pretreated solid tumours. However, their use is associated with adverse effects such as cholecystitis and cholangitis [6, 12], which added complexity to this case. Initially, the recurrent acute cholecystitis was attributed to gallstones rather than an immune-related reaction or metastasis, highlighting the need for a high index of suspicion in evaluating immune-related events, as they can mask or coexist with other conditions. The patient recovered well after laparoscopic cholecystectomy and resumed immunotherapy and was alive and well 8 months later. This highlights the importance of individualised management, particularly when treatment-related toxicities overlap with coexisting pathologies.

## Conclusion

This case highlights the rare metastatic potential of malignant pleural mesothelioma to the peritoneum and gallbladder, emphasising the necessity for histopathological evaluation in atypical presentations of cholecystitis. Additionally, it highlights the diagnostic challenges posed by immune checkpoint inhibitors, which can overlap with coexisting cholecystitis and mimic or obscure disease progression.

## Data sharing

Case report, no additional data to share.
